# Solitary Fibrous Tumor Presenting as Mediastinal Tumor Receiving Blood Supply from Left Internal Thoracic Artery Graft of Coronary Artery Bypass Grafting: A Case Report

**DOI:** 10.70352/scrj.cr.26-0397

**Published:** 2026-07-28

**Authors:** Satona Tanaka, Toyofumi Fengshi Chen-Yoshikawa, Hiroshi Date

**Affiliations:** 1 Department of Thoracic Surgery, Kyoto University Hospital, Kyoto, Kyoto, Japan; 2 Division of Thoracic Surgery, Duke University Medical Center, Durham, NC, USA

**Keywords:** solitary fibrous tumor, mediastinal tumor, coronary artery bypass grafting

## Abstract

**INTRODUCTION:**

A solitary fibrous tumor (SFT) in the thorax typically originates from the visceral pleura, and its presentation as a mediastinal tumor is rare. We report a case of an SFT presenting as a mediastinal tumor with a history of coronary arterial bypass grafting (CABG), in which the left internal thoracic artery (LITA) graft was anastomosed to the left anterior descending artery (LAD), and the LITA graft provided blood flow to the tumor.

**CASE PRESENTATION:**

A man in his 30s was referred to our department for the diagnosis and treatment of an anterior mediastinal mass. He had a history of CABG during childhood, in which the LITA graft was anastomosed to the LAD for a complication of the coronary artery of Kawasaki disease. The tumor was a well-defined anterior mediastinal mass measuring 8.7 cm in size, which was adjacent to the ascending aorta and chest wall but did not show invasiveness. A thymoma or low-grade mediastinal tumor was highly suspected on imaging. Evaluation of the coronary arteries and the graft demonstrated that the LITA graft provided a large amount of blood flow to the tumor, and the right coronary artery provided collateral flow to the LAD area. It is acceptable for the LITA graft to be sacrificed during surgery because of this collateral flow. Surgical resection via re-sternotomy and anterolateral left thoracotomy was performed. The tumor was successfully dissected from the surrounding tissue, and the pedicle of the tumor was recognized at the left upper lobe of the lung. Pathologically, the tumor was diagnosed as SFT without aggressive characteristics.

**CONCLUSIONS:**

SFT presenting as a mediastinal tumor is rare, and impressive imaging of blood supply from the CABG graft was observed. Detailed coronary imaging contributed to the surgical strategy.

## Abbreviations

CABG
: coronary artery bypass grafting
CCTA
: coronary CTA
CD34
: Cluster of Differentiation 34
FDG
: fluorodeoxyglucose
LAD
: left anterior descending artery
LCX
: left circumflex artery
LITA
: left internal thoracic artery
RCA
: right coronary artery
SFT
: solitary fibrous tumor
STAT6
: Signal Transducer and Activator of Transcription 6

## INTRODUCTION

SFT is a relatively rare mesenchymal tumor.^1,2)^ Most thoracic SFTs arise from the visceral pleura, and presentation as a mediastinal tumor is rare.^1,2)^ We report a case of an SFT presenting as a mediastinal tumor in a patient with a history of CABG, where impressive imaging of blood flow from the CABG graft to the tumor was observed. The LITA graft was anastomosed to the LAD, and the LITA graft provided significant blood flow to the tumor, while the RCA provided collateral flow to the LAD area.

## CASE PRESENTATION

A man in his 30s was referred to Kyoto University Hospital for the diagnosis and treatment of an anterior mediastinal mass. In his 10s, CABG was performed for a complication of the coronary artery of Kawasaki disease. In CABG, the LITA graft was anastomosed to the LAD. Enhanced CT revealed a well-defined anterior mediastinal mass measuring 8.7 cm with heterogeneous enhancement, adjacent to the ascending aorta and chest wall, but without apparent invasion (**Fig. 1**). FDG-PET/CT revealed low FDG uptake in the tumor (**Fig. 2A** and **2B**). MRI revealed a well-defined mass lesion with T1-low and T2-mildly high intensity (**Fig. 2C** and **2D**). Based on these findings, a thymoma or low-grade mediastinal tumor was highly suspected. The tumor was found to be attached to the LITA graft; thus, a detailed evaluation of the LITA graft and coronary arteries was performed. CCTA did not show apparent stenosis in the LITA graft or other coronary arteries than the LAD (**Fig. 3**). Coronary angiography surprisingly demonstrated that there was a significant blood supply from the LITA graft to the tumor (**Fig. 4A**). The native LAD was not well visualized on LITA angiography (**Fig. 4B**). The native LAD was hypoplastic and stenotic. The LCX and RCA did not show any stenosis (**Fig. 4C** and **4D**). The RCA provided a good collateral flow to the LAD area (**Fig. 4D**). The LITA can be sacrificed during surgery because of the good collateral flow from the RCA to the LAD area.

**Fig. 1:**
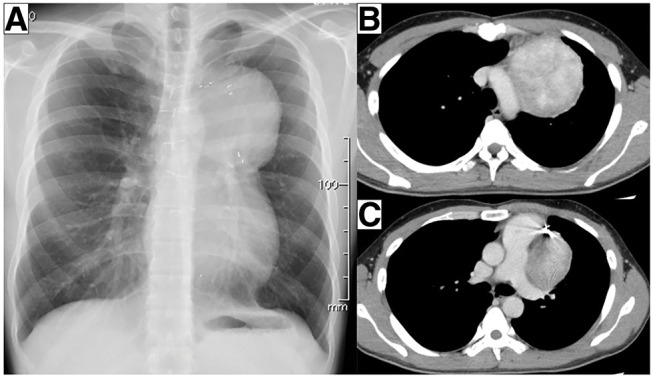
Chest radiography revealed a large mass in the mediastinum with clips used for CABG (**A**). Enhanced CT revealed a well-defined anterior mass measuring 8.7 cm with heterogeneous enhancement (**B**, **C**). Clips for harvesting CABG graft were also identified. CABG, coronary artery bypass grafting

**Fig. 2:**
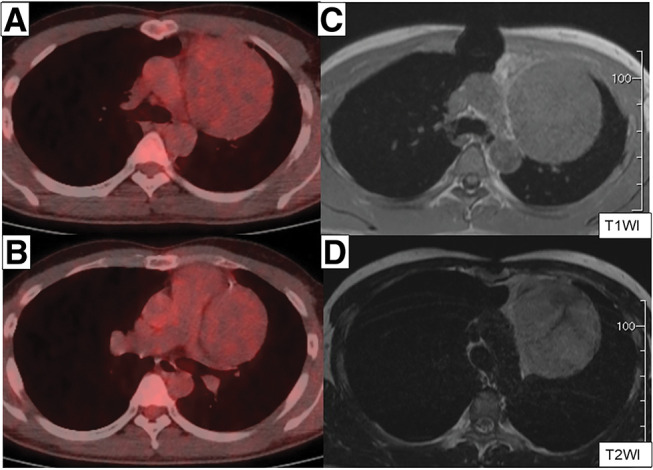
Low FDG uptake was observed on FDG-PET/CT (**A**, **B**). MRI revealed a capsulized mass with T1-low and T2-mildly high intensity (**C**, **D**). FDG, fluorodeoxyglucose; T1WI, T1-weighted intensity; T2WI, T2-weighted intensity

**Fig. 3:**
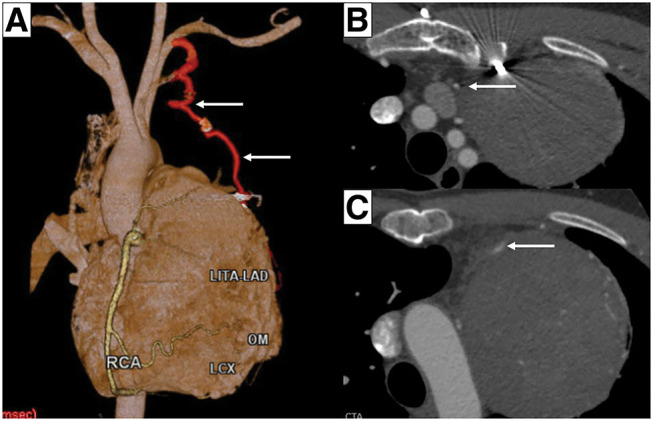
CCTA showed no stenosis of the LITA graft (**A**), and the location of the LITA graft and its association with anterior chest wall and tumor was recognized (**B**, **C**). White arrows indicated the LITA graft. CCTA, coronary CTA; LAD, left anterior descending artery; LCX, left circumflex artery; LITA, left internal thoracic artery; OM, obtuse marginal branch; RCA, right coronary artery

**Fig. 4:**
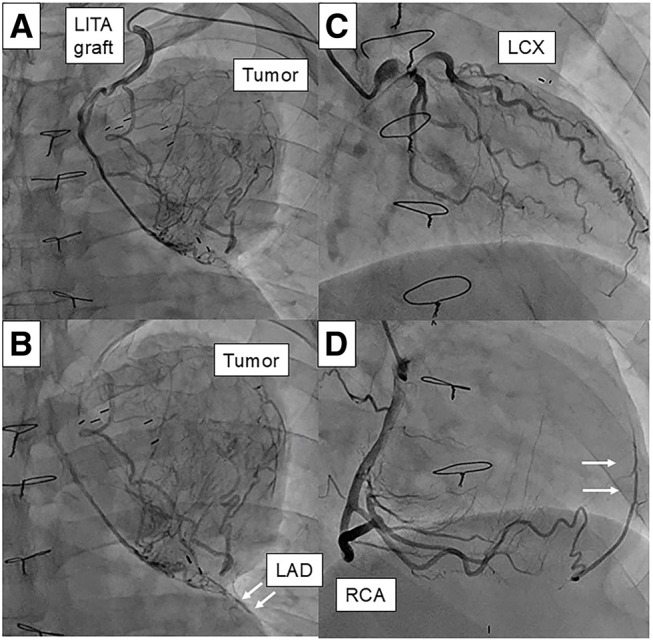
Coronary angiography revealed significant blood supply from the LITA graft to the tumor (**A**), and the LAD was not well visualized by LITA angiography (arrows) (**B**). No stenosis was observed in the LCX (**C**) or RCA (**D**). The RCA provided a good collateral flow to the LAD area (arrows) (**D**). LAD, left anterior descending artery; LCX, left circumflex artery; LITA, left internal thoracic artery; RCA, right coronary artery

We planned surgical resection via re-sternotomy and left thoracotomy, if necessary. The anterior approach was chosen owing to the potential for significant adhesions surrounding the tumor, as well as the possible need for cardiopulmonary support. The anatomical relationship between the LITA graft and the tumor was recognized on CCTA (**Fig. 3B** and **3C**), which would be preserved in the median sternotomy and anterolateral thoracotomy because mediastinal fat tissue was recognized between the LITA graft and the anterior chest wall, and the LITA graft was not close to the anterolateral thoracotomy site. The internal thoracic vein was not recognized on imaging because of artifacts caused by the clips used to harvest the LITA graft during prior CABG. Under general anesthesia, the patient was placed in the supine position, and re-sternotomy was performed by the cardiac surgery team. An anterolateral thoracotomy through the 3rd intercostal space was required to obtain a good surgical view. The internal thoracic vein was not identified, and the intercostal tissue was divided using a vessel sealing system. The tumor was encapsulated and did not invade any major arteries. We began the dissection close to the lung to avoid injury to the LITA graft and feeding vessels, based on their locations recognized by CCTA (**Fig. 3**) and coronary angiography (**Fig. 4**). The mediastinal tissue and visceral pleura were successfully dissected from the tumor at the hypovascular layer (**Fig. 5**). Although most tumor dissections were performed manually and bluntly, massive bleeding was not observed. The LITA graft and feeding arteries were not identified during the resection. The pedicle of the tumor was recognized in the left upper lobe of the lung and dissected using a vessel sealing system, LigaSure (Medtronic, Mansfield, MA, USA), suggesting that the tumor originated from the visceral pleura. The operation time was 177 min, and blood loss was 280 mL. There was no change in the electrocardiogram before and after the resection. The postoperative coronary angiography was not performed. The postoperative course was uneventful, and the patient was discharged on POD 7. Movies of coronary angiography and surgery are provided in **Supplementary Movie 1** and **Supplementary Movie 2**.

**Fig. 5:**
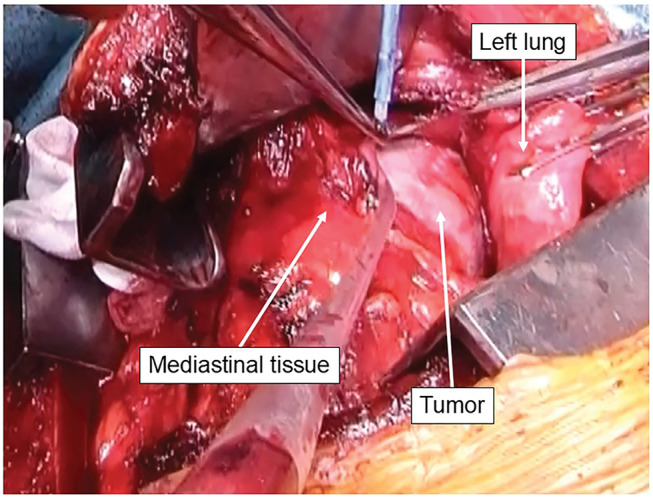
Operative findings showed that the tumor was dissected from mediastinal tissue and left lung. The LITA graft was not identified in the mediastinal tissue. Dissected layer was hypovascular. LITA, left internal thoracic artery

Pathologically, the tumor was diagnosed as SFT. The tumor was encapsulated and a bundle-like proliferation of spindle cells was observed. The spindle cells were positive for CD34 and STAT6, which are key markers for the diagnosis of SFT (**Fig. 6**).^2)^ The Ki-67 labeling index was 5.3%. Signs of aggressiveness, such as necrosis and high mitotic counts, were not observed.^3)^ Three years after surgery, the patient was doing well without any signs of recurrence.

**Fig. 6:**
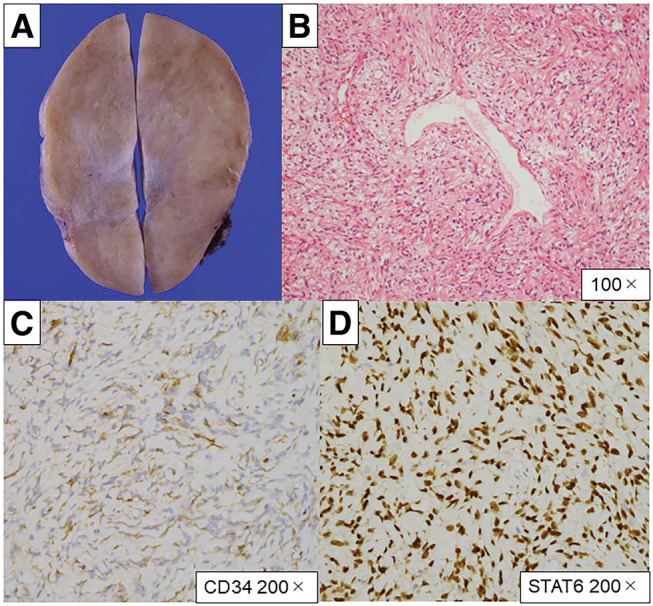
Macroscopically, the tumor was capsulized, and a dissected fibrous pedicle from the lung was observed (arrows) (**A**). Microscopically, bundle-like proliferation (**B**) of CD34-positive (**C**) and STAT6-positive (**D**) spindle cells was observed. CD34, Cluster of Differentiation 34; STAT6, Signal Transducer and Activator of Transcription 6

## DISCUSSION

SFT is a relatively rare mesenchymal tumor that can occur almost anywhere in the body.^1,2)^ On imaging, SFTs typically appear as well-defined, predominantly hypervascular masses with varying degrees of cystic change and necrosis, which usually show heterogeneous enhancement after intravenous contrast administration.^1,2,4)^

Regarding thoracic SFTs, 80% of the tumors originate from the visceral pleura and 20% from the parietal pleura.^2)^ They present with various clinical manifestations, such as large tumors, unexpectedly rapid progression, and hemothorax.^5–7)^ In rare cases, they occur in the lung parenchyma, trachea, and mediastinum.^4,8–10)^ Complete resection provides an excellent prognosis if the tumor does not carry the pathological signs of aggressiveness.^3,11,12)^

The optimal strategy for the safe resection of a large SFT with a rich vascular supply remains unestablished. Manual dissection can be performed along the tumor capsule during SFT resection. On the other hand, the effectiveness of preoperative catheter embolization has been reported.^13)^ Two-stage resection, in which a small thoracotomy for ligation of the feeding vessels is followed by the main tumor resection through a larger surgical window, has also been reported.^14)^ In our case, impressive imaging, in which the tumor presented as a mediastinal mass with high vasculature provided by the LITA graft of past CABG, was noticed, and detailed imaging of the LITA graft and coronary arteries was essential for planning an operative strategy. The LITA graft was patent, but the patency of native coronary arteries other than the LAD and sufficient collateral flow from the RCA to the LAD area were observed; thus, intraoperative ligation of the LITA graft in case of intraoperative bleeding would be acceptable. Preoperative embolization of the LITA graft might be a choice; however, we did not employ this strategy to avoid any unpredicted impact on the heart before surgery.

Regarding the surgical approach, we chose the anterior approach using re-sternotomy and anterolateral thoracotomy rather than posterolateral thoracotomy. Because our preoperative diagnosis was a mediastinal tumor rather than SFT, we needed to consider the potential for significant adhesions surrounding the tumor and the requirement for cardiopulmonary support. The optimal surgical strategy should be discussed on a patient-by-patient basis in such rare cases. Intraoperatively, the tumor was encapsulated and safely dissected from the surrounding tissues based on anatomical understanding of the LITA graft, tumor, and surrounding tissues. The reason why we did not encounter massive bleeding during tumor dissection is that we dissected the hypovascular layers of the capsule. Pathological evaluation showed that the tumor was completely encapsulated, suggesting that the capsule might contain multiple layers, ranging from hypervascular to hypovascular layers. The LITA graft should be preserved based on intraoperative findings; however, the lack of postoperative coronary angiography is a limitation of this case. Interestingly, the tumor had a fibrous pedicle in the visceral pleura. These findings suggest that the SFT originated from the visceral pleura and extended to the mediastinum, obtaining blood supply from the LITA graft, which shows the unique characteristics of SFT regarding the origin of the tumor and the acquisition of blood supply.

To our knowledge, this is the first case report describing an SFT in which blood flow to the tumor was supplied by a CABG graft. Cases of middle mediastinal tumors where blood flow to the tumor was supplied by the coronary artery have been reported.^15–17)^ Coincidentally, all of them were paragangliomas, which are known for their hypervascularity. The hypervascular nature of the tumor may indicate that the tumor vascularization originated from the arteries in the mediastinum, such as the coronary arteries and CABG grafts. Tumor resection was performed under cardiopulmonary support, and 1 patient required concomitant CABG. Another interesting case series is the resection of lung cancer after CABG.^18,19)^ Regardless of tumor invasion, it was difficult to dissect and identify the CABG graft, as experienced in our case. These reports also highlighted the importance of careful preoperative evaluation of coronary arteries, CABG grafts, and their association with tumors.

## CONCLUSIONS

We report a rare case of SFT presenting as a mediastinal tumor, in which the blood supply from the LITA graft of CABG was observed. A detailed evaluation of imaging, including the bypass graft and coronary arteries, contributed to the surgical strategy.

## SUPPLEMENTARY MATERIALS

Supplementary Movie 1 Coronary angiography. The native LAD was hypoplastic and stenotic. LITA angiography revealed a significant blood supply from the LITA graft to the tumor. The native LCX and RCA did not exhibit stenosis. The RCA provided a good collateral flow to the LAD area.
LAD, left anterior descending artery; LCX, left circumflex artery; LITA, left internal thoracic artery; RCA, right coronary artery

Supplementary Movie 2 Surgical video. Re-sternotomy and anterolateral thoracotomy through the 3rd intercostal space were performed to obtain a good surgical view. The tumor was successfully dissected from the mediastinal tissue, mainly using blunt dissection. The stalk of the tumor was recognized in the left upper lobe of the lung and dissected using a vessel sealing system.

## DECLARATIONS

### Availability of data and materials

Data sharing is not applicable to this article because no datasets were generated or analyzed during the study.
